# Supplementary material for: Declines in HIV incidence among men and women in a South African population-based cohort
Source: Nat Commun. 2019 Dec 2;10:5482. doi: 10.1038/s41467-019-13473-y (PMC6889466; doi:10.1038/s41467-019-13473-y)
Supplement: Supplementary file 1 — Supplementary Information [file 41467_2019_13473_MOESM1_ESM.pdf]

Declines in HIV incidence among men and women in a South African population-based cohort

Alain Vandormael[1,2,3,4*], Adam Akullian[5], Mark Siedner[1,6,7], Tulio de Oliveira[4,8,9], Till Bärnighausen[1,3,10], and Frank Tanser[1,2,9,11]

[1]Africa Health Research Institute (AHRI), Private Bag X7, Durban 4013, South Africa.
[2]School of Nursing and Public Health, University of KwaZulu-Natal (UKZN), Durban, 4041, South Africa.
[3]Heidelberg Institute for Global Health (HIGH), University of Heidelberg, Heidelberg, 69120, Germany.
[4]KwaZulu-Natal Research Innovation and Sequencing Platform (KRISP), UKZN, Durban, 4013, South Africa.
[5]Institute for Disease Modelling, Seattle, WA 98005, USA.
[6]Division of Infectious Diseases, Department of Medicine, Massachusetts General Hospital, Boston, MA 02114, USA.
[7]Harvard Medical School, Boston, MA 02115, USA.
[8]College of Health Sciences, UKZN, Durban, 4013, South Africa.
[9]Centre for the AIDS Programme of Research in South Africa (CAPRISA), Durban, 4013, South Africa.
[10]Department of Global Health and Population, Harvard T.H. Chan School of Public Health, Boston, MA 02115, USA.
[11]College of Social Science, University of Lincoln, Lincoln, LN6 7TS, United Kingdom.
[*]Corresponding author: vandormaela@ukzn.ac.za

Supplement Figures

Figure 1: Shows that the proportion of adults that tested for HIV by age and sex remained relatively stable over time.

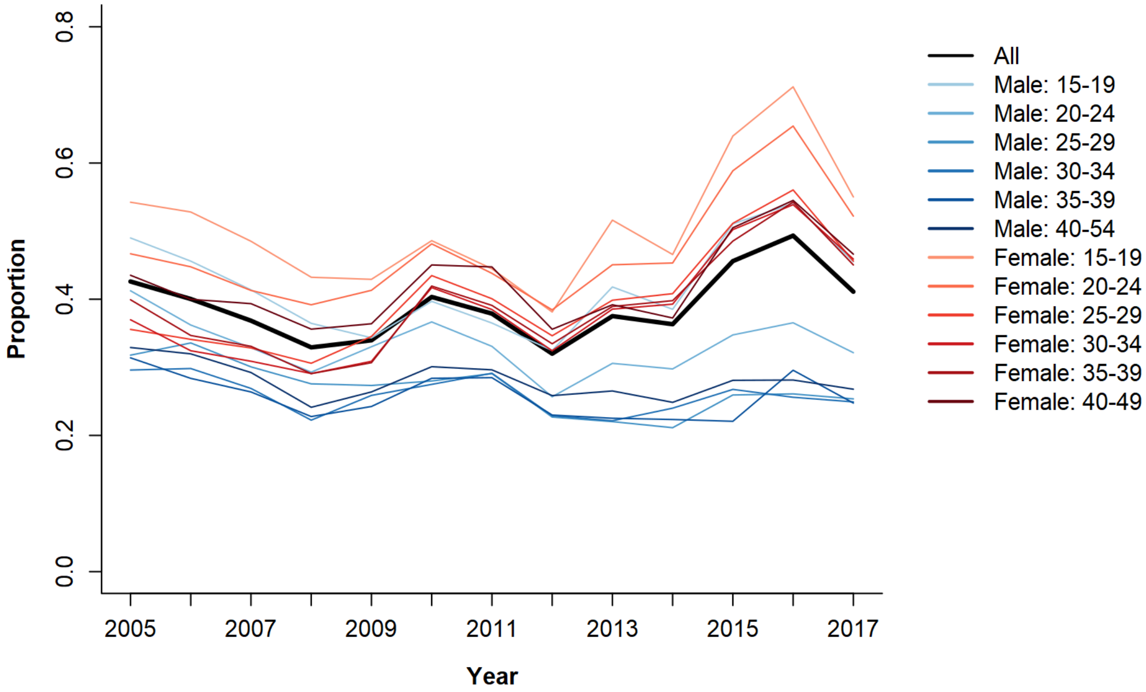

Figure 2: Figure shows that the key demographic characteristics of the HIV testers remained stable over time. Below is the percentage of all HIV-negative testers that were women, the percentage of all repeat-testers (HIV cohort) that were women, the mean age (in years) of all HIV-negative testers and all repeat testers by sex, and the mean number of in-migration and out-migration events among all HIV testers (irrespective of serostatus).

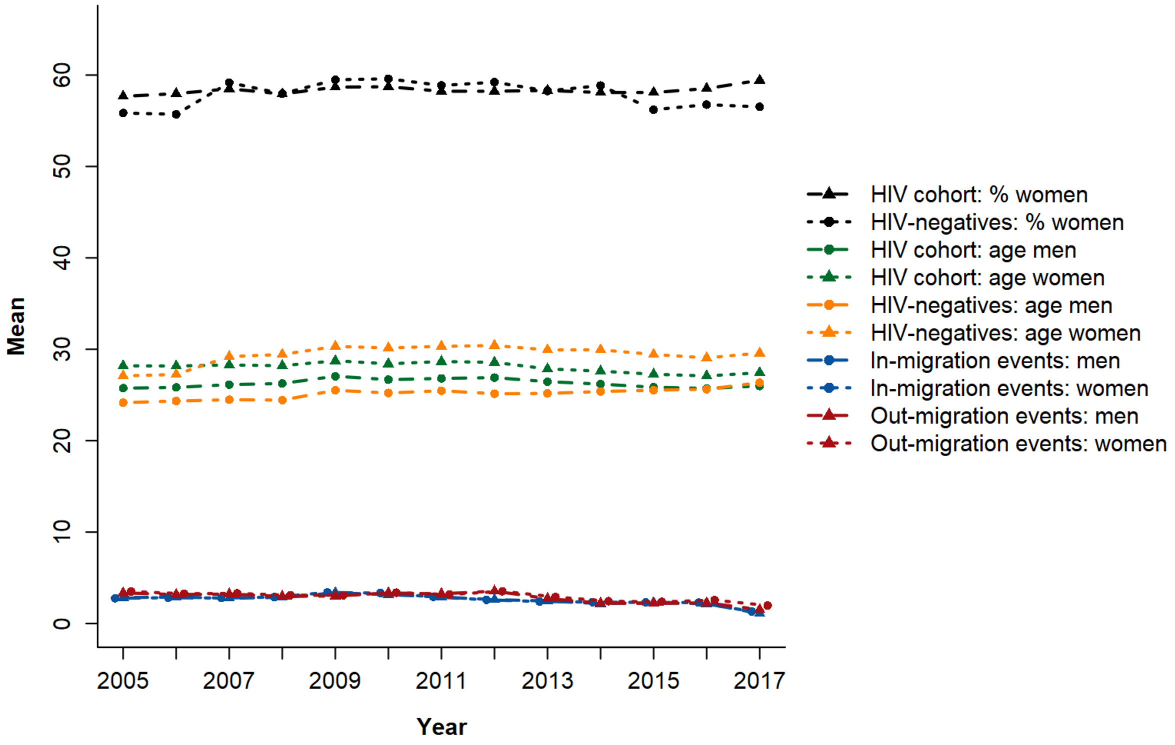

Figure 3: The HIV testing rates and mean age for men reporting being circumcised or not following the roll-out of a voluntary medical male circumcision programme in 2009. The Figure shows little deviation in the mean age between circumcised and uncircumcised men. The HIV testing rates for these two groups mirror the overall HIV testing rate shown in Figure 1.

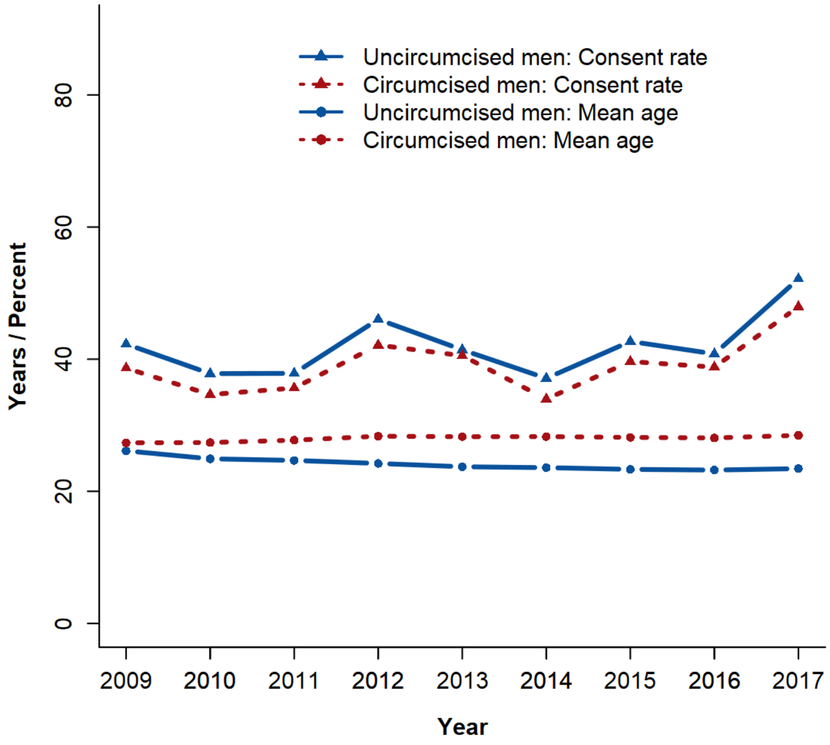

Supplementary Tables

Table 1: Incidence rates (IRs, unadjusted) and incidence rate ratios (IRRs, unadjusted and adjusted) for men and women (N=22,239) by ART coverage, ART scale-up period, and year.

	Events	P-years	Inc. Rate (95% CI)	Unadj. IRR (95% CI)	P-value	Adj. IRR (95% CI)*	P-value
Model 1: By ART coverage[1]							
0–9%	876	25,097	3.49 (3.23–3.77)	Ref.	-	Ref.	-
10–24%	929	25,090	3.71 (3.43–4.00)	1.06 (0.95–1.19)	0.309	0.90 (0.81–1.00)	0.046
25–34%	574	15,147	3.79 (3.43–4.19)	1.09 (0.95–1.24)	0.208	0.86 (0.76–0.97)	0.015
35–55%	1,058	33,329	3.18 (2.97–3.40)	0.91 (0.82–1.01)	0.068	0.66 (0.59–0.75)	<0.01
Model 2: By ART scale-up period[2]							
2005–2010	1,806	50,188	3.60 (3.42–3.78)	Ref.	-	Ref.	-
2011–2015	1,346	37,105	3.63 (3.42–3.85)	1.01 (0.93–1.09)	0.847	0.84 (0.77–0.92)	<0.01
2016–2017	287	11,371	2.52 (2.20–2.89)	0.70 (0.61–0.81)	<0.01	0.58 (0.50–0.67)	<0.01
Model 3: By year[3]							
2005	256	7,807	3.28 (2.81–3.83)	0.83 (0.67–1.04)	0.104	1.21 (1.01–1.46)	0.044
2006	303	8,577	3.54 (3.06–4.08)	0.90 (0.73–1.11)	0.322	1.32 (1.10–1.57)	<0.01
2007	316	8,713	3.62 (3.14–4.18)	0.92 (0.74–1.14)	0.454	1.07 (0.90–1.29)	0.445
2008	318	8,729	3.64 (3.17–4.19)	0.93 (0.75–1.14)	0.475	1.10 (0.92–1.31)	0.314
2009	306	8,301	3.69 (3.18–4.28)	0.94 (0.75–1.17)	0.566	0.99 (0.83–1.18)	0.911
2010	304	8,059	3.78 (3.26–4.37)	0.96 (0.78–1.19)	0.700	1.12 (0.95–1.33)	0.178
2011	284	7,788	3.65 (3.14–4.24)	0.93 (0.74–1.17)	0.515	1.03 (0.87–1.22)	0.717
2012	290	7,358	3.94 (3.37–4.60)	Ref.	-	Ref.	-
2013	279	7,388	3.78 (3.23–4.41)	0.96 (0.76–1.21)	0.724	0.93 (0.78–1.10)	0.391
2014	267	7,376	3.63 (3.09–4.25)	0.92 (0.73–1.16)	0.483	0.95 (0.80–1.13)	0.575
2015	224	7,193	3.11 (2.63–3.69)	0.79 (0.63–1.00)	0.046	0.71 (0.59–0.86)	<0.01
2016	174	6,386	2.73 (2.26–3.29)	0.69 (0.54–0.89)	<0.01	0.69 (0.57–0.84)	<0.01
2017	112	4,984	2.25 (1.79–2.83)	0.57 (0.43–0.75)	<0.01	0.55 (0.44–0.69)	<0.01

[1]ART coverage is for men and women combined, with 0–9% as the reference category. [2]Time intervals were defined by changes in national criteria for ART eligibility, with 2005–2010 as the reference period. [3]Time intervals were defined by year, with 2012 as the reference year. *Estimates adjusted for age, self-reported condom use, marital status, household assets index, cumulative time spent outside surveillance area, and HIV prevalence in the surrounding community (see Table 6 for full results).

Table 2: ART coverage, self-reported condom use, and self-reported circumcision among men.

	**ART coverage**			**Condom use**			**Circumcision**		
Year	N	Perc.	95% CI	N	Perc.	95% CI	N	Perc.	95% CI
2005	1,056	1.5	(0.9–2.4)	3,967	43.6	(42.1–45.2)			
2006	1,186	3.8	(2.8–5.0)	3,706	50.5	(48.9–52.1)			
2007	1,303	8.6	(7.1–10.2)	2,514	52.9	(51.0–54.9)			
2008	1,394	13.6	(11.8–15.5)	2,085	60.3	(58.1–62.4)			
2009	1,477	17.9	(15.9–19.9)	1,919	58.5	(56.3–60.7)	3,381	3.0	(2.4–3.6)
2010	1,638	21.4	(19.5–23.5)	2,083	63.7	(61.6–65.8)	4,096	3.5	(3.0–4.2)
2011	1,725	25.4	(23.4–27.5)	2,044	71.6	(69.6–73.6)	4,406	5.1	(4.5–5.8)
2012	1,777	30.2	(28.0–32.4)	1,254	75.4	(73.0–77.8)	4,373	8.2	(7.4–9.1)
2013	1,893	32.5	(30.4–34.6)	1,496	72.5	(70.1–74.7)	4,160	15.0	(13.9–16.1)
2014	1,993	33.7	(31.6–35.8)	1,365	73.7	(71.3–76.0)	3,513	18.9	(17.6–20.3)
2015	2,128	37.1	(35.1–39.2)	1,456	71.1	(68.7–73.4)	3,903	28.2	(26.8–29.6)
2016	2,299	37.4	(35.4–39.4)	1,439	65.2	(62.7–67.7)	3,515	32.9	(31.3–34.4)
2017	2,580	38.4	(36.5–40.3)	1,367	68.0	(64.3–71.7)			

Table 3: ART coverage among women and male partner condom use reported by women.

	**ART coverage**			**Condom use**		
Year	N	Perc.	95% CI	N	Perc.	95% CI
2005	2,748	2.1	(1.6–2.7)	7,002	33.8	(32.7–34.9)
2006	3,115	5.5	(4.7–6.3)	6,391	41.3	(40.1–42.5)
2007	3,473	10.4	(9.4–11.5)	5,208	44.4	(43.1–45.8)
2008	3,760	14.8	(13.7–16.0)	4,265	50.1	(48.6–51.6)
2009	4,013	19.3	(18.1–20.5)	4,377	46.5	(45.0–48.0)
2010	4,512	24.6	(23.3–25.8)	4,066	51.0	(49.5–52.6)
2011	4,796	30.6	(29.2–31.9)	3,509	60.5	(58.8–62.1)
2012	4,967	35.6	(34.3–37.0)	2,194	62.5	(60.4–64.5)
2013	5,259	39.8	(38.5–41.2)	3,272	63.6	(61.9–65.2)
2014	5,533	43.5	(42.2–44.8)	3,012	62.6	(60.8–64.3)
2015	6,000	48.6	(47.4–49.9)	3,191	64.0	(62.3–65.6)
2016	6,540	49.3	(48.1–50.6)	4,422	58.5	(57.1–60.0)
2017	7,868	50.6	(49.5–51.7)	4,201	68.8	(65.0–72.7)

Table 4: Table shows the adjusted incidence rate ratios (IRRs) by female ART coverage, ART scale-up period, and year for men (N=9,630).

	**Model 1**		**Model 2**		**Model 3**	
	**Adj. IIR (95% CI)**	**P-value**	**Adj. IIR (95% CI)**	**P-value**	**Adj. IRR (95% CI)**	**P-value**
Female ART coverage:						
0–9%	Ref.	-				
10–24%	0.72 (0.58–0.89)	<0.01				
25–34%	0.64 (0.46–0.89)	<0.01				
35–55%	0.52 (0.40–0.68)	<0.01				
ART period:						
2005–2010			Ref.	-		
2011–2015			0.75 (0.62–0.90)	<0.01		
2016–2017			0.52 (0.38–0.72)	<0.01		
Year:						
2005					1.50 (1.04–2.16)	0.028
2006					1.46 (1.02–2.08)	0.038
2007					1.06 (0.74–1.52)	0.755
2008					0.98 (0.68–1.41)	0.910
2009					1.10 (0.78–1.55)	0.599
2010					1.06 (0.76–1.48)	0.732
2011					0.92 (0.65–1.30)	0.621
2012					Ref.	-
2013					0.92 (0.65–1.30)	0.621
2014					0.65 (0.44–0.96)	0.029
2015					0.65 (0.44–0.95)	0.027
2016					0.70 (0.47–1.04)	0.074
2017					0.39 (0.23–0.67)	<0.01
Age (vs. 15–19 years):						
20–24	2.85 (2.24–3.62)	<0.01	2.81 (2.22–3.57)	<0.01	2.81 (2.21–3.57)	<0.01
25–29	4.15 (3.21–5.36)	<0.01	4.05 (3.13–5.23)	<0.01	4.07 (3.15–5.26)	<0.01
30–34	3.28 (2.41–4.46)	<0.01	3.25 (2.39–4.42)	<0.01	3.29 (2.42–4.47)	<0.01
35–54	1.89 (1.41–2.55)	<0.01	1.87 (1.39–2.53)	<0.01	1.90 (1.41–2.55)	<0.01
Circumcised (vs. uncircumcised)	0.58 (0.47–0.71)	<0.01	0.58 (0.47–0.71)	<0.01	0.59 (0.48–0.73)	<0.01
Not married (vs. married)	1.29 (0.96–1.73)	0.093	1.30 (0.97–1.74)	0.081	1.32 (0.98–1.77)	0.065
Household assets (vs. middle):						
lower tertile	0.79 (0.66–0.96)	0.015	0.79 (0.65–0.95)	0.013	0.80 (0.66–0.97)	0.020
higher tertile	0.93 (0.77–1.12)	0.423	0.93 (0.77–1.12)	0.436	0.92 (0.77–1.11)	0.398
Sometimes condom-use (vs. never)	0.94 (0.81–1.10)	0.473	0.93 (0.80–1.09)	0.387	0.94 (0.80–1.10)	0.436
Out-migration (vs <2%):						
2–20%	1.07 (0.88–1.30)	0.522	1.06 (0.87–1.29)	0.555	1.13 (0.92–1.38)	0.240
>20%	1.19 (0.98–1.45)	0.072	1.18 (0.97–1.43)	0.098	1.22 (1.01–1.48)	0.044
Female HIV prev. (vs <25%):						
25–40%	1.47 (1.17–1.86)	<0.01	1.35 (1.08–1.69)	<0.01	1.47 (1.16–1.86)	<0.01
>40%	1.80 (1.34–2.42)	<0.01	1.64 (1.24–2.16)	<0.01	1.90 (1.41–2.56)	<0.01

Inverse probability weights were used to adjust for participant selection and drop-out.

Table 5: Table shows the adjusted incidence rate ratios (IRRs) by male ART coverage, ART scale-up period, and year for women (N=12,609).

	**Model 1**		**Model 2**		**Model 3**	
	**Adj. IIR (95% CI)**	**P-value**	**Adj. IIR (95% CI)**	**P-value**	**Adj. IRR (95% CI)**	**P-value**
Male ART coverage:						
0–9%	Ref.	-				
10–24%	0.99 (0.89–1.11)	0.891				
25–34%	0.87 (0.77–0.97)	0.016				
35–55%	0.68 (0.59–0.78)	<0.01				
ART period:						
2005–2010			Ref.	-		
2011–2015			0.85 (0.77–0.93)	<0.01		
2016–2017			0.63 (0.55–0.74)	<0.01		
Year:						
2005					1.11 (0.90–1.37)	0.339
2006					1.16 (0.95–1.42)	0.157
2007					1.18 (0.97–1.44)	0.097
2008					1.13 (0.92–1.38)	0.234
2009					1.14 (0.94–1.39)	0.176
2010					1.15 (0.94–1.40)	0.169
2011					0.97 (0.79–1.20)	0.803
2012					1.02 (0.83–1.25)	0.846
2013					1.00 (0.82–1.21)	0.968
2014					Ref.	-
2015					0.86 (0.71–1.06)	0.164
2016					0.78 (0.63–0.97)	0.026
2017					0.65 (0.51–0.83)	<0.01
Age (vs. 15–19 years):						
20–24	1.30 (1.17–1.44)	<0.01	1.30 (1.17–1.44)	<0.01	1.29 (1.17–1.44)	<0.01
25–29	1.24 (1.09–1.41)	<0.01	1.24 (1.09–1.41)	<0.01	1.24 (1.09–1.40)	<0.01
30–34	0.92 (0.77–1.09)	0.328	0.92 (0.77–1.09)	0.316	0.92 (0.77–1.09)	0.317
35–54	0.38 (0.32–0.45)	<0.01	0.38 (0.32–0.45)	<0.01	0.38 (0.32–0.45)	<0.01
Not married (vs. married)	1.51 (1.31–1.75)	<0.01	1.51 (1.31–1.74)	<0.01	1.51 (1.31–1.75)	<0.01
Household assets (vs. middle):						
lower tertile	0.99 (0.90–1.10)	0.897	0.99 (0.90–1.10)	0.892	0.99 (0.90–1.10)	0.891
higher tertile	0.93 (0.83–1.03)	0.160	0.93 (0.83–1.03)	0.160	0.93 (0.83–1.03)	0.159
Sometimes condom-use (vs. never)	1.10 (1.00–1.20)	0.043	1.09 (1.00–1.19)	0.045	1.09 (1.00–1.19)	0.045
Out-migration (vs <2%):						
2–20%	1.27 (1.14–1.41)	<0.01	1.27 (1.15–1.41)	<0.01	1.29 (1.16–1.43)	<0.01
>20%	1.15 (1.02–1.29)	0.020	1.15 (1.02–1.29)	0.020	1.15 (1.02–1.29)	0.018
Male HIV prev. (vs <10%):						
10–20%	1.12 (0.98–1.29)	0.101	1.12 (0.98–1.28)	0.108	1.12 (0.98–1.29)	0.108
>20%	1.27 (1.09–1.47)	<0.01	1.26 (1.09–1.46)	<0.01	1.26 (1.09–1.47)	<0.01

Inverse probability weights were used to adjust for participant selection and drop-out.

Table 6: Table shows the full results for the adjusted incidence rate ratios (IRRs) by ART coverage, ART scale-up period, and year for men and women (N=22,239).

	**Model 1**		**Model 2**		**Model 3**	
	**Adj. IIR (95% CI)**	**P-value**	**Adj. IIR (95% CI)**	**P-value**	**Adj. IRR (95% CI)**	**P-value**
ART coverage:						
0–9%	Ref.	-				
10–24%	0.90 (0.81–1.00)	0.046				
25–34%	0.86 (0.76–0.97)	0.015				
35–55%	0.66 (0.59–0.75)	<0.01				
ART period:						
2005–2010			Ref.	-		
2011–2015			0.84 (0.77–0.92)	<0.01		
2016–2017			0.58 (0.50–0.67)	<0.01		
Year:						
2005					1.21 (1.01–1.46)	0.044
2006					1.32 (1.10–1.57)	<0.01
2007					1.07 (0.90–1.29)	0.445
2008					1.10 (0.92–1.31)	0.314
2009					0.99 (0.83–1.18)	0.911
2010					1.12 (0.95–1.33)	0.178
2011					1.03 (0.87–1.22)	0.717
2012					Ref.	-
2013					0.93 (0.78–1.10)	0.391
2014					0.95 (0.80–1.13)	0.575
2015					0.71 (0.59–0.86)	<0.01
2016					0.69 (0.57–0.84)	<0.01
2017					0.55 (0.44–0.69)	<0.01
Age (vs. 15–19 years):						
20–24	1.60 (1.45–1.76)	<0.01	1.59 (1.45–1.75)	<0.01	1.59 (1.44–1.75)	<0.01
25–29	1.68 (1.50–1.88)	<0.01	1.67 (1.49–1.87)	<0.01	1.68 (1.50–1.88)	<0.01
30–34	1.31 (1.13–1.51)	<0.01	1.30 (1.13–1.51)	<0.01	1.31 (1.13–1.52)	<0.01
35–54	0.57 (0.49–0.65)	<0.01	0.56 (0.49–0.65)	<0.01	0.57 (0.49–0.65)	<0.01
Male (vs. Female):						
Circumcised	0.24 (0.20–0.29)	<0.01	0.24 (0.20–0.29)	<0.01	0.24 (0.20–0.29)	<0.01
Uncircumcised	0.48 (0.44–0.52)	<0.01	0.48 (0.44–0.52)	<0.01	0.47 (0.43–0.52)	<0.01
Not married (vs. married)	1.39 (1.23–1.58)	<0.01	1.39 (1.23–1.58)	<0.01	1.41 (1.24–1.60)	<0.01
Household assets (vs. middle):						
lower tertile	0.99 (0.91–1.09)	0.870	0.99 (0.90–1.08)	0.768	1.00 (0.91–1.09)	0.987
higher tertile	0.91 (0.83–1.00)	0.052	0.91 (0.83–1.00)	0.063	0.91 (0.83–1.00)	0.060
Sometimes condom-use (vs. never)	1.11 (1.02–1.20)	0.011	1.10 (1.02–1.19)	0.013	1.11 (1.02–1.20)	0.011
Out-migration (vs <2%):						
2–20%	1.11 (0.99–1.26)	0.082	1.10 (0.98–1.24)	0.117	1.12 (0.99–1.26)	0.077
>20%	1.19 (1.08–1.30)	<0.01	1.18 (1.07–1.30)	<0.01	1.19 (1.08–1.31)	<0.01
Overall HIV prev. (vs <20%):						
20–30%	1.21 (1.08–1.35)	<0.01	1.17 (1.05–1.31)	<0.01	1.23 (1.10–1.39)	<0.01
>30%	1.60 (1.39–1.83)	<0.01	1.50 (1.32–1.71)	<0.01	1.65 (1.43–1.90)	<0.01

Inverse probability weights were used to adjust for participant selection and drop-out.

Table 7: Shows the HIV incidence rates, unadjusted IRRs, and the adjusted IRRs for men (N=9,170) and women (N=11,560) with no more than two consecutive missed test dates between the censoring interval.

	**HIV events**	**Person-years**	**Incidence rate (95% CI)**	**Unadj. IRR (95% CI)**	**P-value**	**Adj. IRR (95% CI)**	**P-value**
Males							
2005	70	3,239	2.16 (1.59–2.92)	1.13 (0.72–1.79)	0.591	1.55 (0.95–2.52)	0.078
2006	80	3,568	2.24 (1.68–2.99)	1.08 (0.68–1.70)	0.752	1.49 (0.92–2.42)	0.109
2007	83	3,634	2.29 (1.73–3.03)	1.04 (0.66–1.64)	0.865	1.33 (0.82–2.14)	0.245
2008	86	3,679	2.35 (1.78–3.10)	0.61 (0.36–1.03)	0.062	0.76 (0.44–1.31)	0.321
2009	84	3,484	2.39 (1.79–3.19)	0.90 (0.56–1.45)	0.657	1.05 (0.64–1.71)	0.854
2010	83	3,379	2.43 (1.81–3.28)	0.95 (0.59–1.52)	0.824	0.97 (0.60–1.56)	0.898
2011	75	3,222	2.31 (1.71–3.12)	0.62 (0.36–1.06)	0.080	0.63 (0.37–1.07)	0.087
2012	75	3,039	2.46 (1.83–3.32)	Ref.	-	Ref.	-
2013	68	3,033	2.25 (1.65–3.07)	0.63 (0.37–1.08)	0.096	0.63 (0.37–1.08)	0.093
2014	54	3,046	1.80 (1.25–2.58)	0.55 (0.32–0.96)	0.035	0.53 (0.30–0.93)	0.028
2015	42	2,967	1.43 (0.95–2.15)	0.39 (0.21–0.73)	<0.01	0.37 (0.20–0.69)	<0.01
2016	32	2,602	1.26 (0.78–2.03)	0.36 (0.19–0.69)	<0.01	0.35 (0.18–0.70)	<0.01
2017	20	1,979	1.02 (0.56–1.85)	0.36 (0.17–0.74)	<0.01	0.35 (0.16–0.73)	<0.01
Females							
2005	186	4,566	3.76 (3.10–4.58)	1.09 (0.86–1.39)	0.465	1.39 (1.08–1.79)	0.011
2006	223	5,007	4.06 (3.37–4.88)	1.14 (0.90–1.44)	0.267	1.46 (1.14–1.86)	<0.01
2007	230	5,078	4.15 (3.48–4.96)	0.97 (0.76–1.24)	0.822	1.20 (0.94–1.54)	0.146
2008	231	5,050	4.26 (3.56–5.11)	0.94 (0.73–1.19)	0.591	1.15 (0.89–1.48)	0.278
2009	222	4,818	4.12 (3.39–4.99)	0.96 (0.75–1.22)	0.717	1.13 (0.88–1.45)	0.342
2010	221	4,681	4.35 (3.61–5.25)	0.81 (0.62–1.04)	0.104	0.95 (0.73–1.24)	0.722
2011	210	4,566	4.21 (3.45–5.14)	0.70 (0.53–0.91)	<0.01	0.81 (0.61–1.06)	0.124
2012	212	4,319	4.52 (3.75–5.46)	0.83 (0.64–1.07)	0.144	0.92 (0.71–1.19)	0.528
2013	212	4,354	4.28 (3.51–5.22)	0.83 (0.64–1.07)	0.150	0.88 (0.68–1.14)	0.323
2014	210	4,329	4.35 (3.57–5.29)	Ref.	-	Ref.	-
2015	184	4,226	3.95 (3.24–4.83)	0.83 (0.64–1.06)	0.137	0.83 (0.65–1.07)	0.155
2016	141	3,783	3.45 (2.73–4.35)	0.80 (0.62–1.04)	0.095	0.80 (0.62–1.04)	0.095
2017	92	3,005	2.70 (2.01–3.63)	0.70 (0.52–0.93)	0.015	0.66 (0.49–0.89)	<0.01

This analysis excludes all repeat-testers who missed two or more consecutive HIV tests betwen their latest HIV-negative and earliest HIV-positive test dates. Results show that the male HIV incidence rate began to decline after 2012 and the female HIV incidence rate began to decline after 2014, which is consistent with the findings for all repeat-testers, irrespective of the number of consecutive missed test dates (see Model 3 of Tables 2–3).
